# Complete Republication: Fall Injury while the Parent Is Operating a Bicycle with an Infant in a Baby Carrier

**DOI:** 10.31662/jmaj.2020-0032

**Published:** 2020-09-30

**Authors:** Osamu Nomura, Yusuke Miyazaki, Hirokazu Takei, Mariko Terauchi, Shun Kishibe, Yusuke Hagiwara, Koji Kitamura, Yoshifumi Nishida, Tatsuhiro Yamanaka

**Affiliations:** 1 Division of Pediatric Emergency Medicine, Department of Pediatric Emergency and Critical Care Medicine, Tokyo Metropolitan Children’s Medical Center, Tokyo, Japan; 2 Graduate School of Information Science and Technology, Tokyo Institute of Technology, Tokyo, Japan; 3 Artificial Intelligence Research Center, National Institute of Advanced Industrial Science and Technology, Tokyo, Japan; 4 Ryokuen Children’s Clinic, Yokohama, Japan

**Keywords:** Injury prevention, bicycle-related injury, infant, head injury, legal regulation

## Abstract

**Introduction:**

Pediatric emergency physicians commonly experience cases of infantile trauma as a result of a child falling from the arms or the back of a parent while the parent is operating a bicycle.

**Methods:**

1. We conducted a retrospective case-series study which included children younger than 1 year who were injured after falling from the arms or the back of the parent while the latter was operating a bicycle.

2. We conducted a dynamics experiment by recreating the circumstances of the accident using dummies representing a 6-month-old infant being carried on the back of the mother. We assessed the score of the Head Injury Criterion (HIC) and the maximum impact load on the head of the dummy infant.

**Results:**

1. We found eight injured patients, two of whom required intensive care. One of the latter experienced neurological sequelae.

2. The HIC score and the maximum impact load varied from 7.7 to 17.0 and 2.26 to 3.47 times the reference values for 6-month-old infants, respectively.

**Conclusions:**

Our study revealed that a strong impact on an infant’s head can result in severe head trauma due to the mechanics of the injury type studied. Preventive strategies for the safe transportation of infants are needed.

## Introduction

Medical professionals in pediatric trauma care in Japan sometimes encounter injuries in infants due to falling from a baby carrier (or buckle carrier), which usually occurs while the parent is operating a bicycle while holding the infant in front or on his or her back using a belt ^(1)^. Infants are likely to fall headlong as their head is heavier than their other body parts; moreover, the center of gravity shifts to the head as infants are unable to take actions to prevent injury, such as using their hands to cover their head, during the fall ^(2)^. These facts account for the higher likelihood of infants sustaining severer trauma than the bicycle operator. In fact, fatalities resulting from this type of accident were reported in 2016 ^(3)^.

In 2006 in Japan, a nationwide “Survey of bicycle operation by parents with young children” was administered to some 6,500 parents of kindergarten and daycare-aged children at 80 sites throughout Japan. A stratified, two-step, randomized extraction method was used based on region and population ^(4)^. The survey revealed that about 1% of the respondents routinely operated a bicycle with the child strapped to their back, and almost 90% of these children were aged 1 year or younger. This style of bicycle riding is often used by parents in Japan to carry children who are unable to sit in a child seat. However, at the time, there were no official reports of injuries, domestically or overseas, due to this style of bicycle riding; therefore, the data needed to prevent injuries resulting from this mode of transportation were insufficient.

In the United States, Europe, Australia, and elsewhere, large-scale surveillances of pediatric injuries conducted at the national level since the 1990s have resulted in the implementation of various preventive measures based on the data obtained. These measures constitute the basis of injury-prevention policies in the respective regions ^(5), (6), (7), (8), (9), (10)^. Also in Japan, a similar surveillance began in the 2000s and has led to the creation of a pediatric injury database containing clinical information collected from multiple medical institutions ^(11), (12), (13)^. However, surveillance is limited in its ability to provide the details of the accident scene, thus making it difficult to recreate the scene in order to formulate injury prevention strategies. To address this issue, researchers have conducted biomechanical experiments using a dummy or *via* simulations based on the accumulated clinical data ^(14), (15)^. In the present study as well, we simulated the probable circumstances of the target injuries based on the available clinical data using a dummy held in the front or on the back of the bicycle operator in order to formulate preventive measures, elucidate the characteristics of craniofacial injuries, and collect mechanical data pertaining to this mechanism of injury.

## Materials and Methods

### 1. Case-series study

#### Study design and the participant selection

We conducted our case review between April 2014 and March 2016 using the craniofacial injury database of the Pediatric Emergency Department at the Tokyo Metropolitan Children’s Medical Center (TMCMC), which includes information such as demographic data (e.g., age and sex), final diagnosis, imaging examination, and disposition of patients younger than 19 years who visited the TMCMC due to head injury. We first selected patients younger than 1 year and then performed a chart review to examine the mechanism of each injury. This procedure allowed us to identify craniofacial injuries resulting from an accident due to holding the infant in the front or on the back of a bicycle operator and to collect mechanical data relevant to these injuries.

#### Data collection

We collected data on the patients’ age, detailed mechanism of injury, bicycle operator, passenger, position of the infant, diagnosis, and Abbreviated Injury Scale (AIS) score, which indicates the anatomical severity of the injured organ, disposition, length of hospital stay, and outcomes. The AIS score was retrospectively evaluated by a chart review.

#### Ethical considerations

This study was approved by the ethics committee of the TMCMC (H28b-41). Patient consent was generally obtained on an opt-out basis *via* the TMCMC website, and written consent was obtained from two hospitalized patients from whom a detailed description of their clinical course was required.

### 2. Accidental fall simulation

#### Experimental method

**Figure 1.**
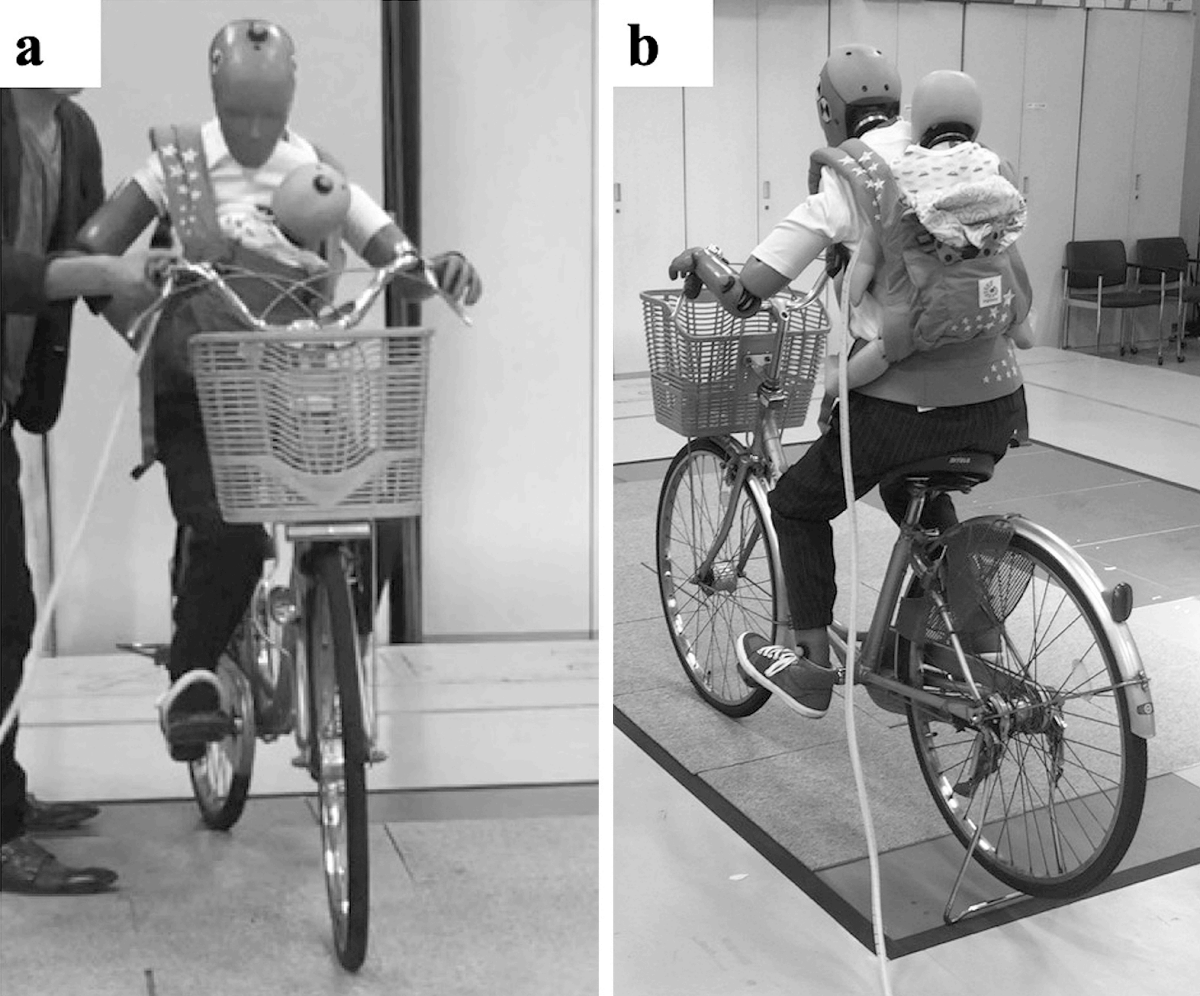
a. Fall experiment with infant held in the front position ^(32)^ b. Fall experiment with infant held in the back position ^(32)^ The permission of using this figure has been obtained from the publisher of the original article (Nomura O, Miyazaki Y, Takei H, et al. Fall injury while the parent is operating a bicycle with an infant in a baby carrier [Hogosha no Jitensha ni Komoritai wo Mochiite DōJōshita Nyūji no Gaishō]. J Jpn Pediatr Soc. 2019;123(5):839-48 ^(32)^).

Bicycle accident simulation was conducted using an anthropometric dummy. A bicycle was “operated” by a small dummy representing an adult woman (Hybrid III AF05), and an infant dummy representing a 6-month-old infant (CRABI 6 month) was held either in the front (Figure 1a) or on the back (Figure 1b) using a baby carrier. A researcher pushed the bicycle forward at a running speed to simulate an accident due to loss of balance occurring at a relatively low velocity. When the displacement of a marker installed on the head of the adult dummy was measured by optical motion capture, the initial velocity of the bicycle was 2.64-3.61 km/h. The experiment was conducted in a motion capture room at the Tokyo Institute of Technology equipped with 16 force plates to measure the ground reaction force at the moment of impact.

#### Equipment and evaluation

A 6DOF motion sensor (6DX pro, DTS) was attached to the head of the infant dummy in order to obtain the head injury criterion (HIC) score; the maximum impact force on the head was measured using a ground reaction force plate (Kistler). Simultaneously, the movement of the dummy at the time of the head impact was recorded *via* sequential photography using a high-speed camera (HAS-D3, Ditect).

The HIC is a frequently used index of the risk of head injury in traffic accidents and was introduced in 1971 by the National Highway Traffic Safety Administration (NHTSA) ^(16)^. An increase in the HIC score (more than AIS4 in severity) reflects an increase in the risk of head trauma and is therefore frequently used in the performance evaluation of automobiles and helmets ^(17), (18)^. The HIC score is calculated based on the linear acceleration of the head *a* and time duration *t* using the following equation:


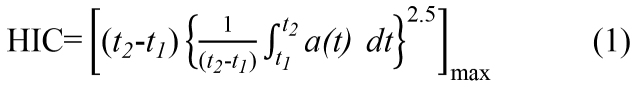


Here, *t_1_* and *t_2_* denote the initial and final times, respectively, of the interval when the HIC value is at its maximum. We assumed that the length of the interval during which the direct force acts on the head was relatively short and evaluated the HIC value at the time of 15 ms from the impact. Previous studies reported a standard HIC value of 390 for a dummy representing a 6-month-old infant ^(19)^.

The maximum impact force, expressed in newton (N), is the maximum force applied to the head at the moment of impact, a significant factor in the mechanism of skull fractures and therefore the most appropriate measure for risk assessment of head injuries ^(20)^. Since the threshold value of the maximum impact force for skull fractures in a 6-month-old infant has not been previously determined, we converted the skull fracture risk curve based on the maximum impact force in adult cadaver experiments to that for a 6-month-old infant based on the scaling rule following the adjustment for bone stiffness and other variables as follows:


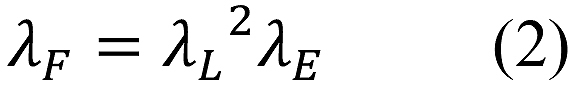


Here, *λ*_F denotes the maximum impact force ratio; *λ*_L, the head dimension ratio; and *λ*_E, Young’s modulus ratio for the skull ^(19), (20), (21)^. For the scaling rule in the performance requirements for a dummy representing a 6-month-old infant, it was reported in a previous study that the head dimension ratio and Young’s modulus ratio were 0.775 and 0.283, respectively. As the maximum impact force for a 95% fracture risk was estimated to be 15,132 N based on Yoganandan’s skull fracture risk curve for the adult skull ^(20)^, the maximum impact load for a 95% risk of a skull fracture was calculated to be 2,569 N after the scaling based on Equation (2).

## Results

### 1. Case-series study

Of the 76,099 patients who visited our emergency department (ED) during the study period, 770 were children with a head injury who were younger than 1 year. We finally identified eight cases of injury which occurred when the child was held in the front or on the back of the bicycle operator (Table 1). Six of these patients were diagnosed with a minor head injury and were discharged from the hospital, whereas the remaining two patients required hospitalization for further treatment.

**Table 1.** Patients in the Case Series ^(32)^.

Age (month)	Injury	AIS (Head)	Outcome	Length of hospital stay	Sequelae	Bicycle operator	Position of other children riding together	Circumstance of injury	Collision with other vehicles	Method of carrying infant
3	Epidural hematoma, parietal bone fracture	2	ICU	3 days	−	Mother	Front and back seats	Traveling	Bicycle	Front
5	Subdural hematoma, cerebral contusion, parietal bone fracture	3	ICU	13 days	Partial paralysis	Mother	Back seat	Traveling	Car	Back
5	Minor head injury	1	Discharged	−	−	Mother	Back seat	When dismounting	−	Front	
6	Minor head injury	1	Discharged	−	−	Mother	Unknown	Traveling	−	Front	
7	Minor head injury	1	Discharged	−	−	Mother	Unknown	Traveling	−	Front	
7	Minor head injury	1	Discharged	−	−	Mother	Unknown	Traveling	Bicycle	Front	
9	Minor head injury	1	Discharged	−	−	Mother	Unknown	Traveling	Bicycle	Front	
10	Minor head injury	1	Discharged	−	−	Mother	Unknown	Traveling	Bicycle	Front	

ICU, intensive care unit; AIS, Abbreviated Injury Scale The permission of using this table has been obtained from the publisher of the original article (Nomura O, Miyazaki Y, Takei H, et al. Fall injury while the parent is operating a bicycle with an infant in a baby carrier [Hogosha no Jitensha ni Komoritai wo Mochiite DōJōshita Nyūji no Gaishō]. J Jpn Pediatr Soc. 2019;123(5):839-48 ^(32)^).

**Figure 2.**
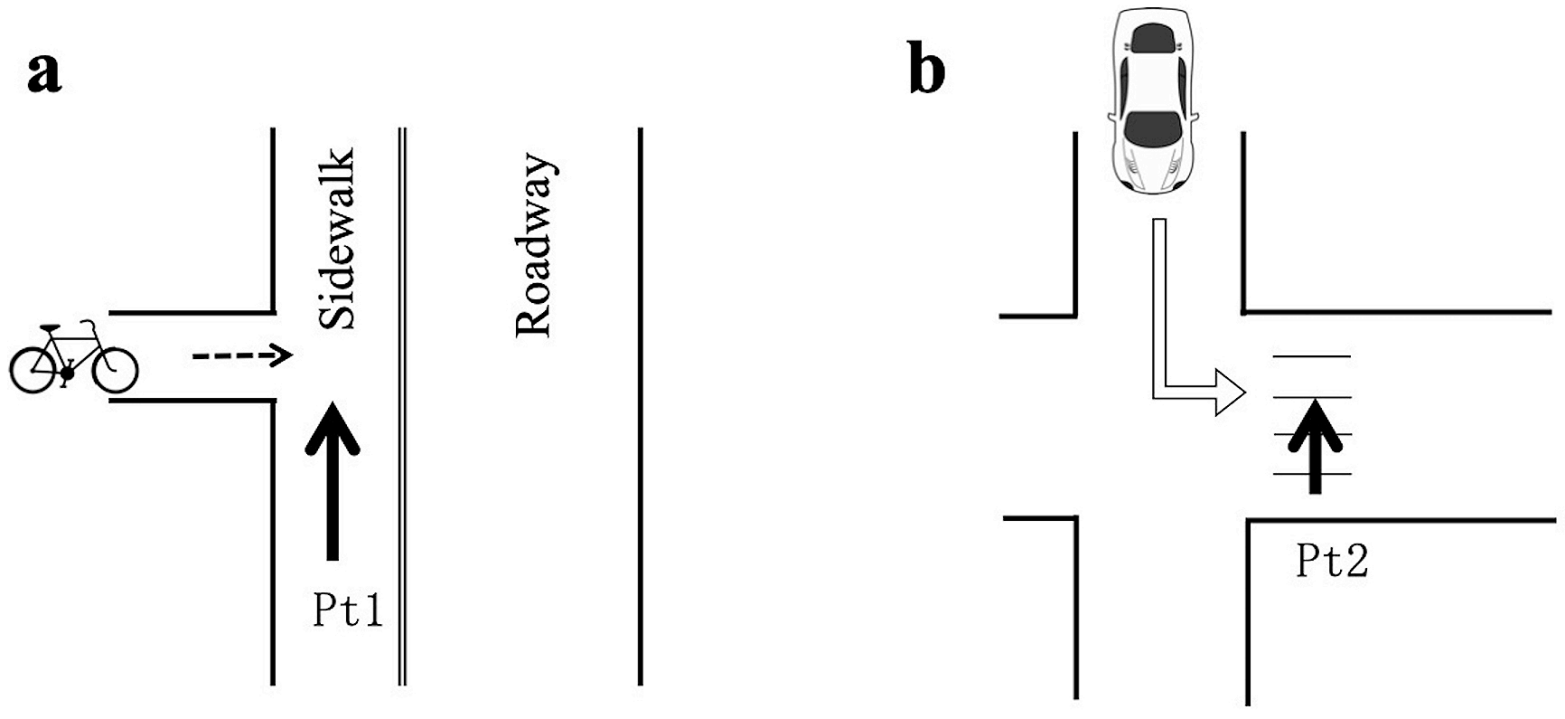
a. Circumstances of injury in hospitalization case 1 ^(32)^ b. Circumstances of injury in hospitalization case 2 ^(32)^ The permission of using this figure has been obtained from the publisher of the original article (Nomura O, Miyazaki Y, Takei H, et al. Fall injury while the parent is operating a bicycle with an infant in a baby carrier [Hogosha no Jitensha ni Komoritai wo Mochiite DōJōshita Nyūji no Gaishō]. J Jpn Pediatr Soc. 2019;123(5):839-48 ^(32)^).

The first hospitalized patient (Figure 2a) was a 3-month-old male whom the mother was holding in her arms using a baby carrier while operating a bicycle with the patient and his siblings (an older brother and an older sister) on the front and back child seats. The mother was traveling on the road with a slight incline to the right side when they were hit by another bicycle coming from the front left. The mother and her children fell on their right side due to the road angle. The mother was able to minimize injury to herself by falling on her right knee. However, she was unable to protect the head of her child, who, on being freed of the baby carrier, struck the right parietal portion of the head against the curb. Upon arrival at our ED, the child was fully conscious, and his head injury was finally diagnosed as a traumatic right epidural hematoma with a parietal bone fracture. His head AIS score was 2, and he was admitted to the intensive care unit (ICU). After careful observation for 3 days in the hospital, he was discharged without any complications.

The second patient (Figure 2b) was a 5-month-old male whom the mother was holding on her back using a baby carrier while operating a bicycle with his older sister on the back seat. When they were crossing the road, an automobile appearing from the front left struck them on their left side, causing them to fall on their right side. The child was freed from the carrier and fell 1 m to the right and front of the bicycle. He struck his head against the road surface. Upon his arrival at the ED by ambulance, his cardiopulmonary state was stable, but he exhibited altered conscious status as indicated by a low score of Glasgow Coma Scale (E3V4M6). A traumatic right subdural hematoma and brain contusion with parietal bone fracture were diagnosed, but the injuries were managed without surgical intervention. His head AIS was 3. He was admitted to the ICU and received 6 days of intensive care. After 13 days of hospitalization, he was discharged with paralysis of the left hand as a complication.

All eight bicycle operators were the patients’ mothers. Among the six discharged patients, the cause of injury in three was a collision with another bicycle, whereas that in the remaining three was loss of balance and falling while riding the bicycle. Only the second hospitalized patient (above) was held on the mother’s back; the other patients were held in their mother’s arms at the time of the accident. Neither of the infants was wearing a helmet. A chart review found that the police came to the scene to manage the aftermath of the accident in these two cases.

### 2. Accidental fall simulation (Table 2)

The experiment was repeated three times with the infant dummy seated in the front and four times with the dummy seated on the back. In the two runs for each scenario, the impact to the infant’s head was determined using a high-speed camera and force plates. The maximum impact forces were found to be 5,801 and 7,103 N when the infant was in the front versus 6,984 and 8,920 N when the infant was on the back. All of the values were 2.26-3.47 times the theoretically estimated value of 2,569 N, corresponding to a 95% risk of skull fracture.

**Table 2.** Fall Experiment ^(32)^.

Method of carrying infant	Attempt	Maximum acceleration [G]	HIC	Maximum impact load [N]	Percentage relative to 95% risk of skull injury (%)
Back	1st	599	6,489	6,984	272
	2nd	632	6,627	Not measurable	Not calculable
	3rd	449	3,901	8,920	347
	4th	404	2,998	Not measurable	Not calculable
Front	1st	61	118	5,801	226
	2nd	58	151	Not measurable	Not calculable
	3rd	40	53	7,103	276

HIC, Head Injury Criteria The permission of using this table has been obtained from the publisher of the original article (Nomura O, Miyazaki Y, Takei H, et al. Fall injury while the parent is operating a bicycle with an infant in a baby carrier [Hogosha no Jitensha ni Komoritai wo Mochiite DōJōshita Nyūji no Gaishō]. J Jpn Pediatr Soc. 2019;123(5):839-48^(32)^).

**Figure 3.**
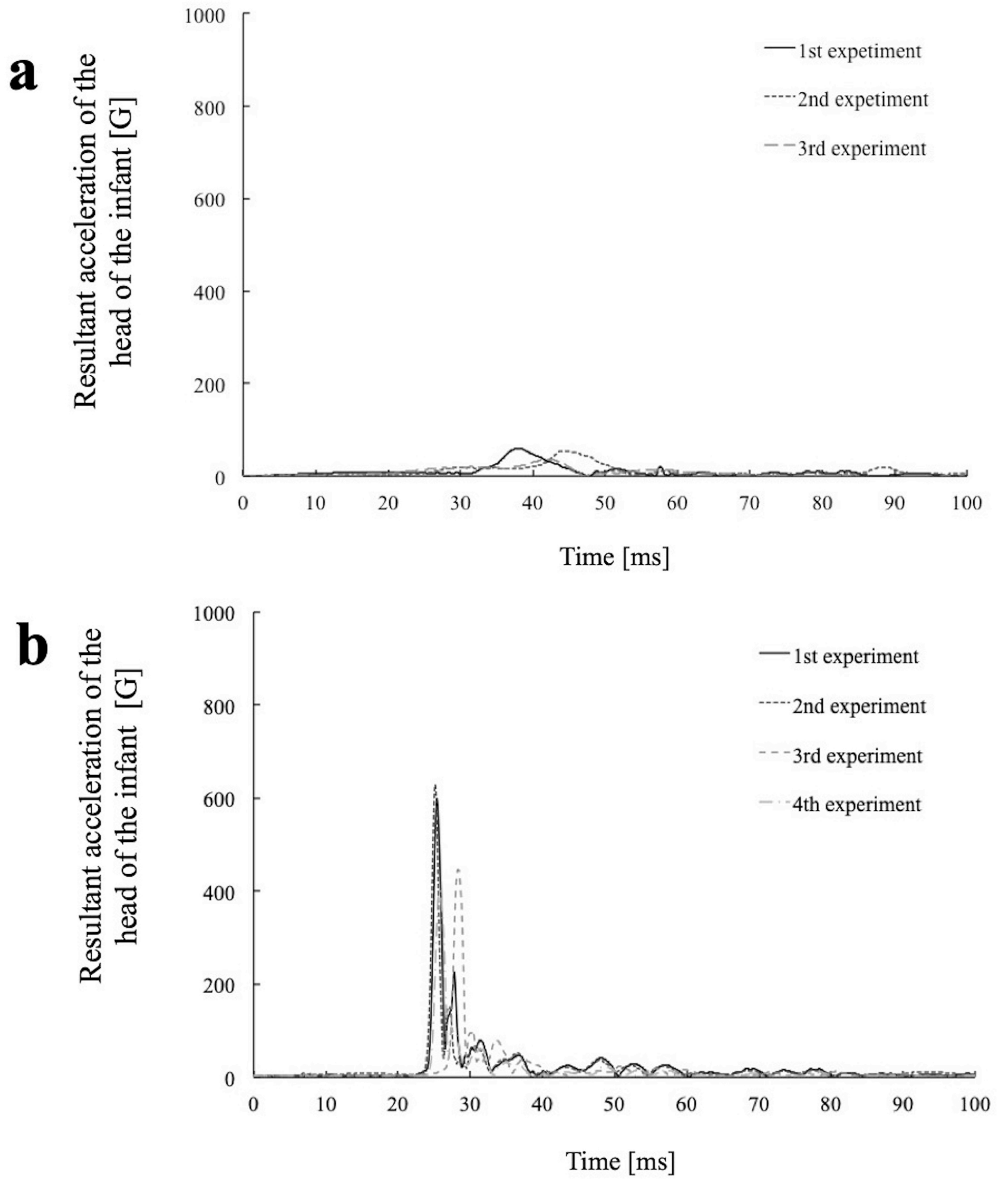
a. Acceleration of the infant’s head in the front position ^(32)^ b. Acceleration of the infant’s head in the back position^(32)^ The permission of using this figure has been obtained from the publisher of the original article (Nomura O, Miyazaki Y, Takei H, et al. Fall injury while the parent is operating a bicycle with an infant in a baby carrier [Hogosha no Jitensha ni Komoritai wo Mochiite DōJōshita Nyūji no Gaishō]. J Jpn Pediatr Soc. 2019;123(5):839-48 ^(32)^).

The HIC score calculated from the acceleration of the infant dummy’s head in the rear position was 2,998-6,627 or 7.7-17.0 times the reference value (390) for a 6-month-old infant. Contrarily, the HIC score in the front position was 53-151, which was remarkably lower than that for the rear position, despite the maximum impact force being comparable in magnitude for the two positions (Figure 3a and b).

**Figure 4.**
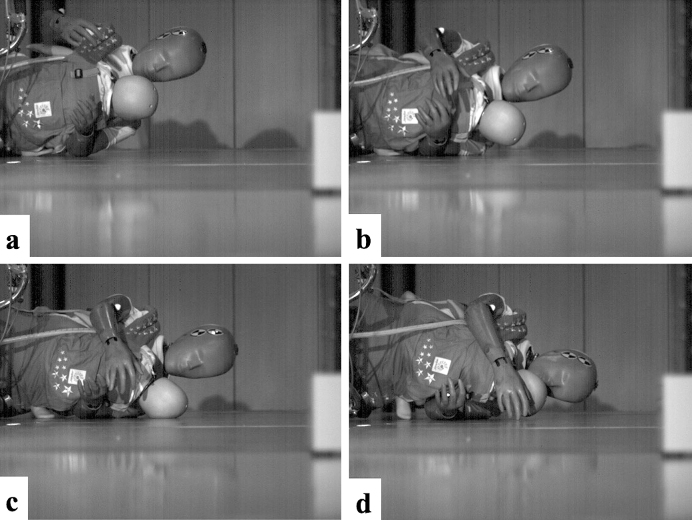
High-speed photos of the infant in the front position. a. Start of acceleration measurement (0 ms) ^(32)^ b. Before head collision (20 ms) ^(32)^ c. At head collision (maximum acceleration) (37 ms) ^(32)^ d. After head collision (60 ms) ^(32)^ The permission of using this figure has been obtained from the publisher of the original article (Nomura O, Miyazaki Y, Takei H, et al. Fall injury while the parent is operating a bicycle with an infant in a baby carrier [Hogosha no Jitensha ni Komoritai wo Mochiite DōJōshita Nyūji no Gaishō]. J Jpn Pediatr Soc. 2019;123(5):839-48 ^(32)^).

**Figure 5.**
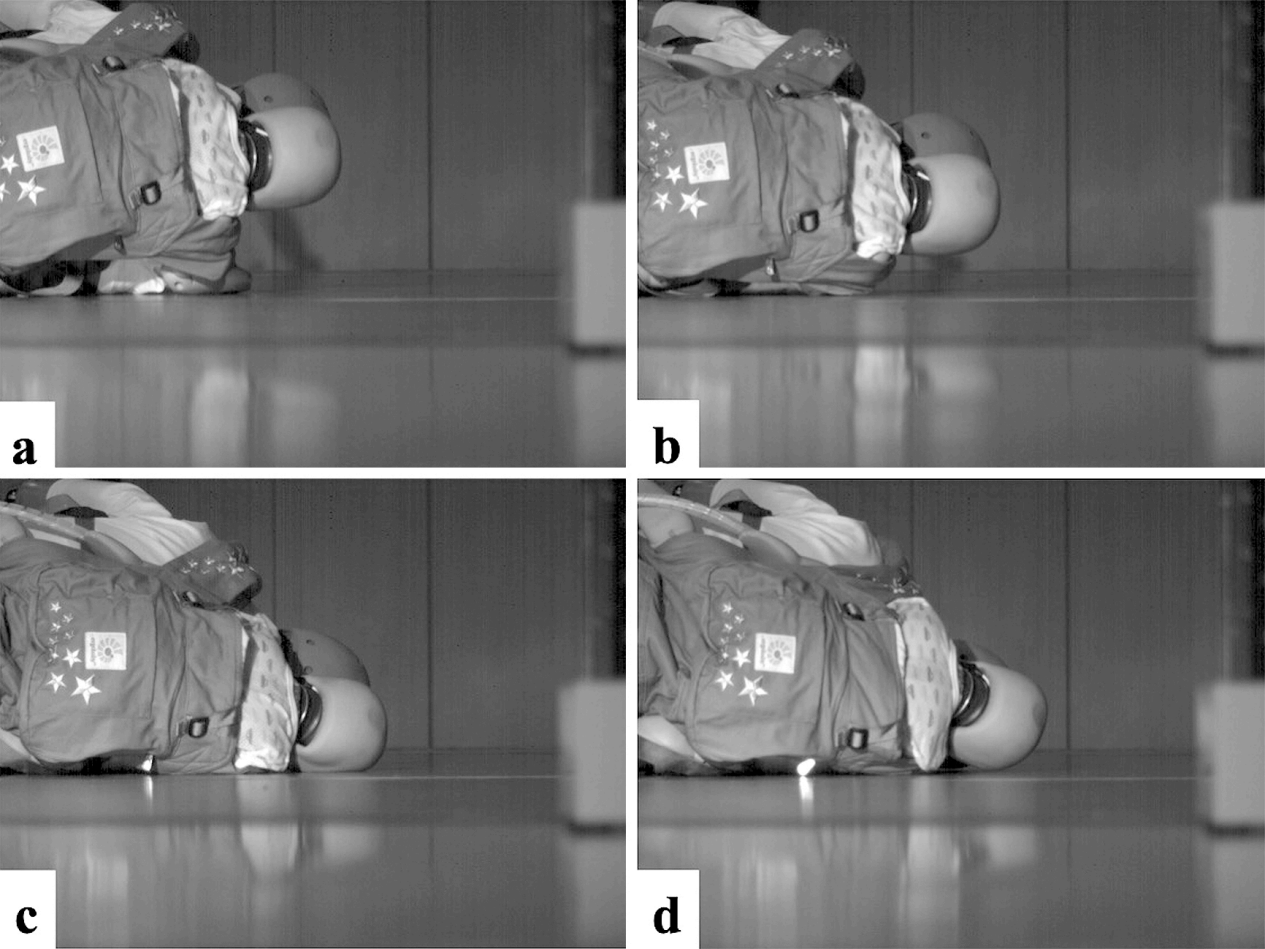
High-speed photos of the infant in the back position ^(32)^ a. Start of acceleration measurement (0 ms) ^(32)^ b. Before head collision (12 ms) ^(32)^ c. At head collision (maximum acceleration) (25 ms) ^(32)^ d. After head collision (37 ms) ^(32)^ The permission of using this figure has been obtained from the publisher of the original article (Nomura O, Miyazaki Y, Takei H, et al. Fall injury while the parent is operating a bicycle with an infant in a baby carrier [Hogosha no Jitensha ni Komoritai wo Mochiite DōJōshita Nyūji no Gaishō]. J Jpn Pediatr Soc. 2019;123(5):839-48 ^(32)^).

The high-speed camera footage revealed that in the front position, the parts of the body that made contact with the ground were the adult’s shoulder, the infant’s head, and the adult’s head in the given order (Figures 4a, 4b, 4c, and 4d). On the infant’s head, the parietal region was the first to hit the ground; it was not deflected from the surface but was instead compressed by its own weight, the carrier belt, or the mother’s arms. Bending of the neck was also observed. When the head was caught between the trunk and the ground, the movement of the head was restricted by its own weight and by the deformation of the neck, both of which reduce the acceleration of the head, resulting in a lower HIC score. Also in the rear position, the adult’s shoulder, the infant’s head, and the adult’s head made contact with the ground surface in the given order (Figure 5a, 5b, 5c, and 5d). The temporal region of the infant’s head hit the ground. The release from the baby carrier and the adult’s body removed impediments from the infant’s trajectory and resulted in a high acceleration and a correspondingly high HIC score.

The maximum impact force on the head was associated with a high incidence of skull fractures in each case. Accordingly, the HIC may not be suitable for skull fracture evaluation in collisions, in which the effective mass volume tends to be increased by the trunk and other factors.

## Discussion

We presented a case series and the results of a fall experiment investigating the features of injuries that occur when an infant is held in the front or on the back of an adult operating a bicycle. Our study found that two of the eight patients required hospitalization, one of whom experienced neurological sequelae. The mechanism of injuries to a child held in the front or on the back of a bicycle rider was explored *via* fall experiments, which revealed that in both positions, the risk of a skull fracture was more than 95%. This study was able to document the mechanism of severe injuries and the magnitude of the forces acting on an infant’s head. While comparatively rare, this type of injury is severe. High-speed photography revealed that the trajectory of the infant dummy held on the back of an adult bicycle operator was unimpeded, causing the infant to be thrown from the baby carrier and its head to hit with the ground, as clinically observed in the most severe cases of head trauma in bicycle-related accidents involving infants. Contrarily, we found that the infant dummy held in the front remained in the arms of the rider.

There are a few studies discussing the mechanisms of pediatric injury in the context of a descriptive case series incorporating the results of biomechanical experiments. In Japan, studies similar to the present one have been conducted to investigate mild injuries occurring at comparatively high frequencies, such as bicycle-spoke injuries and toothbrush-induced intra-oral injuries, in order to improve product safety ^(22), (23), (24)^. In the present study, we revealed the effectiveness of a translational approach combining clinical and experimental biomechanical data in exploring the mechanism of severe injuries.

The “Survey of bicycle operation by parents with young children” conducted in Japan in 2006 ^(4)^, which included items concerning accidents due to loss of balance, found that nearly half the respondents thought they would probably experience an accident due to loss of balance, with about 10% reporting having actually had such an experience. In fact, the respondents’ anxiety about such accidents tended to increase when the child seat was located behind the operator or when a “high-back” child seat was placed at the rear of the bicycle than in other positions (when the child seat was located in front of, or in the conventional position behind, the operator’s seat). This suggests that placing a young child in a child seat in a higher, rear position increases instability during bicycle operation by shifting the center of gravity of the bicycle.

In Japan, bicycle riding by two or more persons is prohibited by the Road Traffic Act ^(25)^. However, the rules of the road traffic vary by prefecture, with some prefectures allowing individuals aged ≥ 16 years to operate a bicycle equipped with seats for two small children or a bicycle with one child in a child seat and another child strapped to the operator’s back with a baby carrier ^(26), (27)^. An infant held on the back of the rider using a baby carrier, however, is regarded as a part of the rider. Furthermore, operating a bicycle with an infant in front of the rider is also permitted, depending on the jurisdiction. Given this state of affairs, parents are commonly seen in Japan riding a bicycle with an infant strapped to his/her front or back.

To prevent injuries resulting from accidents of the sort described, the development of 1) a helmet for infants; 2) a highly adhesive baby carrier; 3) a better-designed child seat for infants; 4) a bicycle trailer for carrying infants; and 5) improved regulations are desirable. The feasibility of these options was investigated *via* SWOT (strengths, weaknesses, opportunities, and threats) analysis (Table 3) ^(28), (29)^.

**Table 3.** SWOT Analysis for Injury Prevention ^(32)^

	Helmet for infants	Highly adhesive baby carrier	Child seat for infants	Bicycle trailers	Passage of regulations
Strengths	Development is feasible based on the current designs for child helmets.	Development is feasible based on the current designs for baby carriers.	Development is feasible based on the current designs for child seats.	Commonly used overseas and minimally affects riding instability	Most effective for injury prevention
Weaknesses	Risk of increasing cervical spine instability and airway compression in infants	Risk of load increase from the parent’s trunk on the infant at the time of the fall	Heavy reinforcement of the device may increase instability during bicycle riding.	Insufficient bicycle roads in Japan	Might deprive parents with a young child of their means of transport (i.e., compensatory strategies are needed)
Opportunities	Potential to develop a new technology for product creation	Potential to develop a new technology for product creation	Potential to develop a new technology for product creation	Potential to develop a new market for this product	Can aid child care if compensatory measures are provided
Threats	Incorrect use of the product can be harmful to infants (e.g., airway compression)	Use of the carrier is complicated. Also, incorrect use may cause harm to infants (e.g., compromised chest wall movement)	Instability may increase the risk of accidents.	Insufficient bicycle parking spaces in Japan	Caregivers’ objections and financial issues

SWOT, strength weakness opportunity threat The permission of using this table has been obtained from the publisher of the original article (Nomura O, Miyazaki Y, Takei H, et al. Fall injury while the parent is operating a bicycle with an infant in a baby carrier [Hogosha no Jitensha ni Komoritai wo Mochiite DōJōshita Nyūji no Gaishō]. J Jpn Pediatr Soc. 2019;123(5):839-48 ^(32)^).

First, developing a helmet for infants based on the current designs for child helmets is not difficult. However, the instability of the cervical spine in infants and issues surrounding helmet-associated airway compression must be addressed. In addition, ethical problems may occur in testing helmets with infants during the manufacture and design process.

Second, developing a highly adhesive baby carrier which can prevent a child from being thrown from a bicycle is also technologically feasible. However, even if such a device were invented, the risk of a load increase from the parent’s trunk on the baby at the time of the fall, as demonstrated by the high-speed camera footage in the fall experiment in this study, remains.

Third, a new child seat that is more heavily reinforced than the existing high-back-type child seat for bicycle use may be developed, but such a device will probably result in further instability during riding, as suggested in the responses in the aforementioned nationwide survey in Japan.

Fourth, bicycle trailers are commonly used overseas and therefore can be easily introduced into Japan. However, research is initially needed to determine the safety of bicycle trailers as well as the availability of sufficient parking spaces, given the fact that Japan currently suffers from a shortage of cycling lanes and bicycle parking spaces.

Finally, passage of regulations prohibiting bicycle riding with an infant or small child in the front or on the back should contribute to the prevention of bicycle-related injuries. Nevertheless, such regulations might deprive parents with infants of their means of transportation and should be weighed carefully to assess their social impact. Compensatory measures might include a reduction or elimination of public transportation fares for such parents. The feasibility of this policy depends to some extent on the scale of economic loss attributable to the type of injury described in this study. In the United Kingdom and New York City, bicycle riding by two or more persons, regardless of age, is prohibited ^(30), (31)^. These legal precedents will hopefully inspire further discussion in Japan.

This study has several limitations: This was a retrospective study based on a review of clinical records at a single center. Selection bias and other biases due to incomplete medical record data may have been introduced. Due to our focus on craniofacial injuries, injuries of the limbs and trunk in the same type of accident were not investigated. Further, the effect of protective reactions by the parent was not considered; our experiment simulated a near-worst-case scenario using dummies. Moreover, neither the reproducibility of the friction between the infant dummy and baby carrier nor the effect of the type of baby carrier and bicycle was evaluated. However, the epidemiologic characteristics in this case-series study demonstrated a tendency similar to that observed in a large-scale, nationwide survey. Thus, this study contributed detailed information on the mechanism of bicycle injuries and medical data. The results of the fall experiment also supported the clinical findings and may have helped to provide further perspectives on the mechanism of these bicycle-related injuries.

The biomechanical forces involved in injuries that occur when an infant is held in the front or on the back of a bicycle operator are enormous and exceed the tolerance values for skull fractures caused by the impact to the infant’s head. Based on these findings, new preventive measures should be considered.

## Article Information

### Conflicts of Interest

None

### Acknowledgement

We thank the physicians of the pediatric emergency department at Tokyo Metropolitan Children’s Medical center for collecting the data for this study, Ms. Yukiko Oishi for managing the data, and Mr. Rui Sugisaki at the Tokyo Institute of Technology for his support in conducting the experiment.

### Author Contributions

Osamu Nomura contributed to the study design, data analysis of the case series, and drafting of the manuscript. Yusuke Miyazaki designed and conducted the experimental study and drafted the manuscript. Hirokazu Takei, Mariko Terauchi, Shun Kishibe, and Koji Kitamura supported the experimental study and supervised the drafting of the manuscript. Yusuke Hagiwara, Yoshifumi Nishida, and Tatsuhiro Yamanaka provided expert advice and supervised the drafting of the manuscript. All the authors have approved the submission of this manuscript.

### Ethical Statement

This study was approved by the ethics committee of the Tokyo Metropolitan Children’s Medical Center (H28b-41).

### Note

This article was based on a study first reported in the *Journal of the Japan Pediatric Society* 2019; 123: 839-848, in Japanese ^(32)^. This paper has been accurately translated into English, The original version is available at http://www.jpeds.or.jp/journal/abstract/123-05.html#123050839. The Editors-in-Chief of *Journal of the Japan Pediatric Society* and* JMA Journal* have permitted the publication of this manuscript.

## References

[1] Committee on Improving Childhood Living Environments of the Japan Pediatric Society. Injury Alert. Severe head injury in an infant resulting from being thrown from the caregiver’s back in a bicycle accident. [No.71. Jitensha Untenchu no Hogosha ni Seowareta Jōtai Kara Tentōji ni Hōshutsu Sare Jūshōtōbugaishō wo Otta Nyūji]. J Jpn Pediatr Soc. 2017;121(7):1277-80.

[2] Bachur RG, Shaw KN. Fleisher & Ludwig's textbook of pediatric emergency medicine. 7th edn. Philadelphia: Lippincott Williams & Wilkins; 2015.

[3] A 7-month-old male patient died due to a collision between a car and a bicycle in Kokubunji when his mother fell while carrying him on her back. Asahi Shinbun. 2016 May 6. Tokyo Honsha-Ban. Yukan. P13.

[4] Survey of bicycle operation by parents with young children. [Yōji no Jitensha-dōjō no Jittai nadoni Kansuru Chōsa Hōkokusho] [Internet]. Japan Bicycle Promotion Institute [cited 2019 Jun 21]. Available from: https://www.jbpi.or.jp/report_pdf/00000081_20060630133545.pdf.

[5] Yamanaka T. Injury surveillance [Jiko no Surveillance]. Jpn J Pediatr. 1998;51(3):418-26.

[6] Injury Prevention Legislation Database [Internet]. National Conference of State Legislatures [cited 2019 Jun 21]. Available from: http://www.ncsl.org/research/health/injury-prevention-legislation-database.aspx.

[7] National Center for Injury Prevention and Control [Internet]. Centers for Disease Control and Prevention [cited 2019 Jun 21]. Available from: https://webappa.cdc.gov/sasweb/ncipc/nfirates.html.

[8] European Injury Data Base [Internet]. European Commission [cited 2019 Jun 21]. Available from: https://ec.europa.eu/health/data_collection/databases/idb_en.

[9] European Detailed Mortality Database [Internet]. World Health Organization Regional Office for Europe [cited 2019 Jun 21]. Available from: https://gateway.euro.who.int/en/datasets/european-mortality-database/.

[10] Authoritative information and statistics to promote better health and wellbeing [Internet]. Australian Institute of Health and Welfare [cited 2019 Jun 21]. Available from: https://www.aihw.gov.au/reports-data/health-conditions-disability-deaths/injury/overview.

[11] Yamanaka T. Tacking injury prevention. [Injury prevention ni Torikumu]. Jpn J Pediatr Med. 2007;39(7):1006-15.

[12] Yamanaka T. Child safety. [Kodomo no Anzen]. J Pediatr Prac. 2008;71(11):1919-21.

[13] Nishida Y, Motomura Y, Yamanaka T. Approach to injury prevention in children. [Kodomo no Shōgai-Yobō eno Approach]. Jpn J Pediatr Med. 2007;39(7):1016-23.

[14] Yamanaka T. Approach to data collection for injury prevention. [Shōgai-Yobō ni Tsunagaru Jōho Shūshū eno Approach]. J Child Health. 2008;67(2):177-90.

[15] Nishida Y. Integrative utilization approach of the multi-organizational distributed data for injury prevention. J Jpn SIDS Res Soc. 2012;12(1):16-25.

[16] Versace J. A review of the severity index. Proceedings of the Stapp Car Crash Conference; 1971; Coronado, CA. New York: SAE paper; 1971. No. 710881.

[17] American Automobile Manufacturers Association. Proposal for Dummy Response Limits for FMVSS 208 Compliance Testing. Attachment C of American Automobile Manufacturers Association. 1998;61-114.

[18] Mertz HJ, Irwin AL, Prasad P. Biomechanical and scaling bases for frontal and side impact injury assessment reference values. STAPP Car Crash Jour. 2003;47:155-88. 17096249 10.4271/2003-22-0009

[19] Melvin JW. Injury Assessment Reference Values for the CRABI 6-Month Infant Dummy in a Rear-Facing Infant Restraint with Air Bag Deployment. SAE Transact. 1995;104(6):1553-64.

[20] Yoganandan N, Banerjee A. Survival analysis-based human head injury risk curves: focus on skull fracture. J Neurotrauma. 2018;35(11):1-8. 29409390 10.1089/neu.2017.5356

[21] Irwin A, Mertz J. Biomechanical basis for the CRABI and Hybrid III child dummies. Proceedings of the 41st Stapp Car Crash Conference; 1997; Lake Buena Vista, FL. New York: SAE paper; 1997. No. 973317.

[22] National Institute of Advanced Industrial Science and Technology. [Heisei 22-nendo Chūshō Kigyō Shien Chōsa Kids Design Seihin Kaihatsu Shien Jigyō Hōkokusho] [Internet]. [cited 2019 Jun 21]. Available from: http://warp.da.ndl.go.jp/info:ndljp/pid/11241027/www.meti.go.jp/policy/mono_info_service/mono/human-design/file/22fyreport/H22fykidsdesign_honbun.pdf.

[23] Standard of Infants’ Chair for Bicycles [Jitensha-you Nyūji Zaseki no SG Kijun] [Internet]. Consumer Product Safety Association [cited 2019 Jun 21]. Available from: https://docs.wixstatic.com/ugd/c4350a_17dbd2787dc64a8c86da15418759ef67.pdf.

[24] Nishida Y, Kitamura K, Oono M, et al. Development of novel toothbrush to prevent penetrating injury of children. Inj Prev. 2016;22(Suppl 2):A291.

[25] Road Traffic Act [Internet]. Article 63-11. e-Gov [cited 2019 Jun 21]. Available from: http://law.e-gov.go.jp/html/data/S35/S35HO105.html.

[26] Tokyo Metropolitan Road Traffic Regulation [Internet]. Article 10. Tokyo Metropolitan Regulation Database [cited 2019 Jun 21]. Available from: http://www.reiki.metro.tokyo.jp/reiki_honbun/g1012199001.html.

[27] Osaka Prefecture Road Traffic Regulation [Internet]. Article 11. Osaka Prefecture Regulation Internet Version [cited 2019 Jun 21]. Available from: http://www.pref.osaka.lg.jp/houbun/reiki/reiki_honbun/k201RG00001084.html.

[28] Zavala DE, Bokongo S, John IA, et al. Implementing a hospital based injury surveillance system in Africa: lessons learned. Med Confl Surviv. 2008;24(4):260-72. 19065866 10.1080/13623690802373884

[29] Lamontagne ME, Swaine BR, Lavoie A, et al. Analysis of the strengths, weaknesses, opportunities and threats of the network form of organization of traumatic brain injury service delivery systems. Brain Inj. 2011;25(12):1188-97. 21939374 10.3109/02699052.2011.608211

[30] Road Traffic Act 1988 Section 24 Restriction of carriage of persons on bicycles [Internet]. Legislation.gov.uk [cited 2019 Jun 21]. Available from: http://www.legislation.gov.uk/ukpga/1988/52/section/24.

[31] Safe Bicycling in New York [Internet]. The official website of the city of New York [cited 2019 Jun 21]. Available from: http://www.nyc.gov/html/dot/downloads/pdf/bicyclerules_english.pdf.

[32] Nomura O, Miyazaki Y, Takei H, et al. Fall injury while the parent is operating a bicycle with an infant in a baby carrier [Hogosha no Jitensha ni Komoritai wo Mochiite DōJōshita Nyūji no Gaishō]. J Jpn Pediatr Soc. 2019;123(5):839-48.

